# Effect of Steroids on Patients With Fibromyalgia/Chromic Widespread Pain: An Observational Study

**DOI:** 10.7759/cureus.53736

**Published:** 2024-02-06

**Authors:** Obuli Srinivasan Gurunathan, Eswaradass Chellapandian, Sibi Thirunavukkarasu, Sathvika Thermalingem, Prasanna Eswaradass

**Affiliations:** 1 Internal Medicine, Mohan Kumaramangalam Medical College, Salem, IND; 2 Neurology, Appusami Hospital, Salem, IND; 3 Neurology, University of Alberta Hospital, Edmonton, CAN; 4 Neurology, University of Saskatchewan, Saskatoon, CAN; 5 Neurology, University of Kansas Health System, Kansas City, USA

**Keywords:** steroids, thyroid autoimmunity, anti-thyroid peroxidase antibodies, pain management, fibromyalgia

## Abstract

Objective: Fibromyalgia causes widespread chronic pain. Pain management and treating underlying conditions are of utmost importance. Recent studies found an association of thyroid autoimmunity with fibromyalgia. Pain management of patients with anti-thyroid peroxidase antibody (anti-TPO Ab) positive was studied sparsely. To determine the effect of steroid (deflazacort) on pain management using numerical rating scale (NRS) pain score at baseline and at 3-month follow-up.

Study design: A retrospective observational study was undertaken, recruiting patients diagnosed with fibromyalgia as per 2010 American College of Rheumatology guidelines and treated with the steroid, deflazacort 12 mg. Patients with missing details were excluded. Patients were categorized into negative, positive, and strongly positive anti-TPO Ab groups. Baseline and follow-up (3 months) pain score was compared across the groups. Reduction in pain was considered as a primary outcome variable.

Results: The study included 128 participants with 98 (76.6%) females and 30 (23.4%) males. The age of the study population was 48±13.29 years. The proportion of hyper, hypo, and euthyroid was 10 (7.81%), 42 (32.81%), and 76 (59.38%), respectively. The proportion of participants with negative, positive, and strongly positive anti-TPO Ab levels was 41 (32.03)%, 50 (39.06%), and 37 (28.91%), respectively. Baseline pain score was 7.3±1.32 and 3-month follow-up was 4.7±2.46. Steroid response was found in 66 (51.6%). Negative and positive anti-TPO Ab had a 1-point reduction in pain score from baseline, p-value <0.001. The strongly positive group had 5 points reduction, p-value<0.05.

Conclusion: Fibromyalgia patients with thyroid autoimmunity responded well to short courses of steroids. Greater pain relief was observed among those who are strongly positive anti-TPO Ab group.

## Introduction

Fibromyalgia (FM) lies in a spectrum of disorders that often lack classification precisely and is considered part of the overview of somatic and functional syndromes [1]. It causes widespread chronic pain with ancillary symptoms and diagnosis is difficult because of varied clinical presentations [2]. FM is often characterized by sleep disturbances, fatigue, and idiopathic pain associated with infections, neurological disorders, diabetes, and rheumatic pathologies [3,4]. FM often presents as a chronic disease significantly impacting quality of life. The prevalence of FM worldwide has reached 2-3% [5]. In India, the prevalence in the general population was around 0.2 to 11.4%[6]. FM imposes a considerable economic burden on the patients. In the USA and Europe, it incurs an estimated annual cost of 1750 to 35920$ and 1250 to 8504$, respectively [7]. As of now, there are no biomarkers available to diagnose FM and its diagnosis is purely clinical. In 1990, the American College of Rheumatology came up with the clinical diagnostic criteria. As per this criterion, the patient is said to be diagnosed with FM clinically if the patient has chronic widespread pain for 3 or more months and is accompanied by 11 or more out of 18 bilateral tender points [8]. The etiology of FM is unclear, however, genetics, family history, and triggers like injury or infection seem to play a role. FM is associated with conditions like osteoarthritis, ankylosing spondylosis, and rheumatoid arthritis. It is more prevalent among the female sex [9]. FM is also found to be associated with medical disorders like mental or psychiatric disorders, gastrointestinal disorders, and neurological disorders [10]. Recent studies found that thyroid autoimmunity has been associated with chronic widespread pain and FM. Anti-thyroid peroxidase antibodies (anti-TPO Ab) were seen to be elevated in the patients with FM [11]. However, levels of anti-TPO Ab were not found to be significantly associated with the severity of symptoms [12]. Treatment of FM often relies on pain management and treating the underlying condition. Treatments include pharmacological measures like serotonin-noradrenaline reuptake inhibitors (SNRIs), tricyclic antidepressants (TCA), and gabapentinoids. Some non-pharmacological measures like exercise, education, and behavioral therapy are also helpful in certain cases [13]. Recent studies emphasized the autoimmune cause of widespread pain among FM patients, however, they fail to mention the treatment for FM associated with thyroid autoimmunity [14]. Management of FM among the patients with anti-TPO Ab positive was studied sparsely. Hence, the current study was designed to fill this paucity in the literature.

Objectives

To determine the effect of a short course of steroids (deflazacort) among FM patients with anti-TPO Ab-negative, positive, and strongly positive and comparing their pain level at baseline and at 3-month follow-up using numerical rating scale (NRS).

## Materials and methods

A retrospective observational study was carried out at Appusami Hospital, Salem, India for a period of 4 years, that is, from July 2018 to July 2022. All the participants who presented to the outpatient department of neurology and were diagnosed with FM were considered as the study population. Patients diagnosed with FM as per 2010 American College of Rheumatology guidelines were included in the study. The study included all the patients who were treated with deflazacort 12 mg once daily for 2 weeks followed by 6 mg daily for 2 weeks and then 3 mg for 2 weeks, that is, total duration of 6 weeks of steroid taper. The availability of anti-TPO Ab status was the other inclusion criterion. Patients whose details were missing in the medical case records were excluded. All the eligible patients were recruited by convenient sampling by retrospective case record review.

Participants were categorized into negative, positive, and strongly positive anti-TPO Ab groups based on the levels as <15, 16-500, and >500 IU/ml, respectively. All the details like age, gender, levels of anti-TPO Ab, pain as per NRS (out of 10), levels of thyroid-stimulating hormone (TSH), T4 and T3, and past medical history were collected into a predesigned structured data collection sheet. All the patients were followed up at 3 months, and the data corresponding to the pain level was collected. Baseline NRS was compared to NRS at 3-month follow-up. The effect of corticosteroids on pain management across the groups was analyzed.

Reduction in pain was considered as a primary outcome variable. TPO level was considered as a primary explanatory variable. Descriptive analysis was carried out by mean and standard deviation for quantitative variables, frequency, and proportion for categorical variables. Median pain scores were compared between baseline and follow-up within each subgroup of anti-TPO Ab using the Wilcoxon signed-rank test. P-value < 0.05 was considered statistically significant. Data was analyzed by using coGuide REAP software version 1 (BDSS Corp., India) [15].

## Results

A total of 128 subjects were included in the final analysis. The mean age of the patients was 48.00 ± 13.29 years, and 98 (76.56%) of them were females. TPO positivity was noted in 50 (39.06) patients and high TPO titers were found in 37 (28.91) patients. Sixty-six (51.56%) patients had a good steroid response. Forty-two (32.81%) patients had hypothyroidism. The mean NRS for pain at baseline was 7.3 ± 1.32 and at the 3-month follow-up was 4.7 ± 2.46 (Table 1).

**Table 1:** Summary of baseline parameters EU: euthyroid; TPO: thyroid peroxidase; TSH: thyroid-stimulating hormone

Parameters	Summary
Age (in years)	48.00 ± 13.29
Age group	
Up to 35	23 (17.97%)
36 to 55	69 (53.91%)
>55	36 (28.13%)
Gender	
Female	98 (76.56%)
Male	30 (23.44%)
TPO level	
Negative (<15)	41 (32.03%)
Positive (15 to 500)	50 (39.06%)
Strongly positive (>500)	37 (28.91%)
TSH level	
<0.27	9 (7.03%)
Normal (0.27 to 4.2)	59 (46.09%)
>4.20	60 (46.88%)
T3 level	
<0.8	20 (15.63%)
0.8 to 2.0	97 (75.78%)
>2.0	11 (8.59%)
T4	8.24 ± 3.33 (range 0.90 to 19.70)
<5.10	15 (11.72%)
5.10 to 14.10	108 (84.38%)
>14.10	5 (3.91%)
Steroid response	66 (51.56%)
Thyroid status	
Hyper	10 (7.81%)
Hypo	42 (32.81%)
EU	76 (59.38%)
Pain scale at baseline	7.3 ± 1.32 (range 5 to 10)
Pain scale At 3-month follow-up	4.7 ± 2.46 (range 0 to 9)

Out of 128 participants, all of them (100%) had polymyalgia, followed by hypothyroidism in about 42 (32.81%), diabetes in 31 (24.22%), hypertension in 28 (21.88%), polyarthralgia in 13 (10.16%), and depression in 10 (7.81%) (Table 2).

**Table 2:** Summary of medical history DM: diabetes mellitus; HTN: hypertension; COPD: chronic obstructive pulmonary disease; ICT: intracranial tension

Medical history	Summary
Polymyalgia	128 (100%)
Hypothyroidism	42 (32.81%)
DM	31 (24.22%)
HTN	28 (21.88%)
Polyarthralgia	13 (10.16%)
Depression	10 (7.81%)
Seizures	7 (5.50%)
Anxiety	6 (4.69%)
Headache	5 (3.91%)
Stroke	5 (3.91%)
COPD	2 (1.56%)
Papilledema	1 (0.78%)
Benign ICT	1 (0.78%)
Goiter	1 (0.78%)

Median (IQR) pain score as per the NRS scale at baseline was 7 (6 to 7.50) among negative TPO Ab group, 7 (6 to 7) among positive TPO Ab group, and 8 (8 to 9) among strongly positive TPO level. Median (IQR) pain score at the 3-month follow-up was 6 (4.50 to 7) among negative TPO Ab group, 6 (3 to 7) among positive TPO Ab group, and 3 (2 to 4) among strongly positive TPO Ab group. The difference in the pain score between baseline and follow-up in the three categories was found to be statistically significant with p values 0.001, <0.001, and <0.001, respectively (Table 3).

**Table 3:** Comparison of median pain scale in baseline and 3-month follow-up across TPO level NRS: numerical rating scale; TPO: thyroid peroxidase

TPO levels	Pain score as per NRS scale		P-value*
	Baseline	Follow-up	
Negative (<15)	7 (6, 7.50)	6 (4.50, 7)	0.001
Positive (15 to 500)	7 (6, 7)	6 (3, 7)	<0.001
Strongly positive (>500)	8 (8, 9)	3 (2, 4)	<0.001

## Discussion

In this retrospective case-cohort study, we included a total of 128 patients of which 67.97% of patients were positive for anti-TPO Ab. We found that 51.6% of the patients responded well to the steroid therapy. We also found that the FM patients who were strongly positive for anti-TPO Ab responded more to steroids than the other groups of patients.

Bazzichi et al., hypothesized in their study that thyroid autoimmunity may play a role in the pathophysiology of FM [16]. The association between thyroid autoimmunity and FM is an area of ongoing research and discussion. While there may be some evidence to suggest a potential link, the relationship is complex and not fully understood. It was found that the severity of FM does not correlate with anti-TPO Ab [17]. The prevalence of FM among patients with Hashimoto’s thyroiditis was found to be 62% in a study [18]. A systematic review and meta-analysis carried out by Park et al. reported significantly higher levels of anti-TPO Ab among FM than controls [11]. In the study conducted by Pamuk and Cakir, the prevalence of anti-TPO Ab among FM was found to be 24.2% [19]. A recent meta-analysis carried out by Park et al. concluded that anti-TPO Ab positivity was more prevalent among FM patients with an OR of 3.41 compared with healthy controls [11]. The prevalence of positive anti-TPO Ab among FM patients was found to be almost 3-fold higher than that reported by a retrospective study carried out in Japan by Nishioka et al., who reported the prevalence as 13.2% [12]. In our study, we found the prevalence of anti-TPO Ab among FM patients was 67.97% which is higher than most of the above studies. This difference in the prevalence could be due to the sample size variation between each of these studies. Such discrepancy in prevalence between studies needs to be further clarified by studying a larger cohort of patients [20]. A retrospective analysis of 5 years of data conducted by Munipalli et al. has reported female predominance over males among the FM population group, which is similar to our study [21].

Aleksi et al., in their cross-sectional study in Finland, reported hypothyroidism in 34% of patients among FM which is similar to our study (32.81%). We did not evaluate the need for thyroxine among FM patients as most of our patients were already diagnosed with hypothyroidism and were on treatment before the diagnosis of FM [22].

Serotonin-norepinephrine reuptake inhibitors (SNRI) like milnacipran and duloxetine, and pregabalin are the FDA-approved drugs for the treatment of FM [23]. A meta-analysis carried out by Häuser et al.’s placebo-controlled randomized control trial studying various antidepressants like SNRI, selective serotonin reuptake inhibitors (SSRI), tricyclic antidepressants (TCA), monoamine oxidase inhibitors (MAOI) reported that TCAs were more effective in improving pain, sleep quality, and fatigue, of which duloxetine is the only one to get FDA approval [24]. A systemic review conducted by Welsch et al. that included double-blinded randomized trials comparing the effect of SNRI with placebo in the management of FM has reported that greater population from the SNRI treating arm has dropped out in comparison to placebo due to various adverse effects (19% SNRI versus 10% placebo) [25]. It is reported that out of every 100 FM patients taking these medications, 13-20 dropped out due to adverse effects [26]. Since most of these medications have minimal pain relief with more adverse effects, we sought to check the efficacy of steroids on FM. Most of our patients had already tried TCA, gabapentinoids without significant benefit in terms of pain relief. In our study, we found that more than half of the patients responded well to short courses of steroids (51.6%). Adverse effects were not a problem since it’s a short course of therapy and as anticipated none of our patients discontinued therapy due to side effects. Dirawi and Habib looked at the effectiveness of intramuscular betamethasone in patients with FM and elevated CRP. They concluded that intramuscular betamethasone is effective for FM symptoms as evidenced by improvement in the FM impact questionnaire (FIQR)[27].

Non-pharmacological therapies like water therapy and acupuncture therapy are also found to be effective in reducing the pain associated with FM [28,29]. A randomized control trial conducted by Guinot et al., to study the effect of repetitive transcranial magnetic stimulation and multicomponent therapy on pain management among patients with FM, reported a significant reduction in pain at 14-week follow-up [30]. Infrastructure availability and the treating physician's choice have a greater part in the choice of this mode of treatment.

The major limitation of this study is that it's a retrospective study with a smaller sample size. Hence there is a high probability of chance findings. Also, selective inclusion of the patients with the availability of anti-TPO Ab status and six weeks of tapering steroid therapy would have introduced natural selection bias. The possibility of reporting and ascertainment bias could not be completely ruled out as both the participants and the investigators were aware of the nature of the treatment. We couldn’t evaluate the role of potential confounding or effect modification due to inadequate sample size.

Currently, FM treatment involves a multidisciplinary approach with a combination of both pharmacological and non-pharmacological measures. Based on our experience we recommend a short course of deflazacort for patients with FM and high titers of anti-TPO Ab. However, these findings need to be confirmed with a larger prospective study. Although FM is not regarded as an autoimmune disease, a strong association with autoimmune thyroiditis raises the possibility of autoimmune etiology. Future studies should focus on understanding the pathophysiology of FM and its causal relationship with anti-TPO Ab.

## Conclusions

Patients with FM with higher anti-TPO Ab titers responded well to a short course of steroid therapy in our study. We suggest a trial of short-course steroid therapy in this particular subset of FM patients. However, the results need to be confirmed and validated with a larger prospective study to establish their reliability and applicability to a broader population. Future research should focus on understanding the underlying pathophysiology of FM and its potential causal relationship with thyroid autoimmunity. There might be a link between the two conditions, and exploring this connection could provide valuable insights for potential future management strategies.
